# Prevalence of Certain Corneal Conditions and their Demographic Risk Factors; Tehran Geriatric Eye Study

**DOI:** 10.34172/aim.28831

**Published:** 2024-08-01

**Authors:** Alireza Hashemi, Hassan Hashemi, Mohammadreza Aghamirsalim, Alireza Jamali, Mehdi Khabazkhoob

**Affiliations:** ^1^Noor Research Center for Ophthalmic Epidemiology, Noor Eye Hospital, Tehran, Iran; ^2^Noor Ophthalmology Research Center, Noor Eye Hospital, Tehran, Iran; ^3^Eye Research Center, Tehran University of Medical Sciences, Tehran, Iran; ^4^Department of Medical Surgical Nursing, School of Nursing and Midwifery, Shahid Beheshti University of Medical Sciences, Tehran, Iran

**Keywords:** Corneal abnormalities, Corneal degeneration, Epithelial defect, Geriatric, Population-based, Posterior embryotoxon, Prevalence

## Abstract

**Background:**

Corneal abnormalities are one of the important reasons for visual impairment. There is little evidence of the prevalence of different types of corneal abnormalities. The aim of this study was to assess the prevalence of various corneal abnormalities and identify the key risk factors associated with these abnormalities in an elderly population residing in Tehran.

**Methods:**

The Tehran Geriatric Eye Study (TGES) was conducted as a cross-sectional study, utilizing a population-based approach and employing stratified cluster random sampling. The study focused on individuals aged 60 years and above residing in Tehran. An ophthalmologist performed a slit lamp examination to evaluate the eyelid, cornea, and crystalline lens.

**Results:**

The prevalence of posterior embryotoxon (PE), punctate epithelial defect (PED), pigment on endothelium (POE), corneal dystrophy (CDys), corneal vascularization (CV), and corneal degeneration (CDeg) were estimated to be 0.08% (95% confidence interval [CI]: 0.02 to 0.40), 8.77% (95% CI: 6.64 to 11.51), 0.57% (95% CI: 0.33 to 0.98), 0.53% (95% CI: 0.33 to 0.82), 0.95% (95% CI: 0.60 to 1.52), and 44.87% (95% CI: 41.80 to 47.98), respectively. Overall, approximately 49.08% of the participants exhibited some form of corneal abnormality in at least one eye. The multiple logistic regression model revealed that increasing age was significantly associated with PED, CV, and CD. Furthermore, illiterate participants had a significantly higher prevalence of PE.

**Conclusion:**

The findings of this study indicate that approximately half of the elderly population aged 60 years and above in Tehran have at least one corneal abnormality, with corneal degeneration being the most prevalent. Age was identified as the primary determinant of corneal abnormalities.

## Introduction

 Corneal abnormalities are a significant contributor to visual impairment and rank as the fifth leading cause of blindness globally, following cataracts, refractive errors, glaucoma, and age-related macular degeneration.^1-3^ Estimates suggest that corneal abnormalities are responsible for 8%-25% of the cases of blindness in developed countries; this is while the major burden of corneal abnormalities is related to developing countries.^2^ Due to the effects of corneal abnormalities on vision^4^ and its high prevalence, 184 576 corneal transplants were done across the world in 2012.^5^ The importance of corneal abnormalities is due to their outcomes; for example, corneal vascularization (CV) results in opacity in the vision path and causes significant visual impairment which sometimes requires corneal transplantation in advanced stages.^6,7^ Furthermore, some corneal abnormalities like posterior embryotoxon (PE) are associated with increased intraocular pressure and the risk of open-angle glaucoma.^8,9^

 The epidemiology of corneal pathologies varies in different studies.^10^ Moreover, there is little evidence on the prevalence of different types of corneal abnormalities; for example, studies have reported prevalence rates of 1.68%^11^ and 27.2%^12^ for corneal opacity and a prevalence of 0.3%^13^ and 0.2%^14^ for corneal dystrophy (CDys) in Iranian and Indian elderly populations, respectively. Also, 897 per one million people were observed to have CDys in the USA.^15^ This variety in the results of different studies is due to different factors including causative factors like age, gender, education level, availability and general standards of eye care, and study population.^3,7,10^ A literature search only found one population-based study addressing the prevalence of different corneal abnormalities in an Iranian elderly population,^13^ although other studies have been conducted to evaluate some corneal abnormalities individually.^16^ Therefore, more epidemiological studies are required in this regard in different age groups and in different societies for proper health policymaking.

 According to projections, the world’s elderly population has expanded significantly due to the improvement in life expectancy. Currently, 11% of the global population is aged 60 years or above, and this percentage is expected to double by 2050, reaching 22%. Iran, as a developing country, exhibits a population aging trend that mirrors the global pattern.^17^ Projections indicate that by the year 2050, the elderly population in Iran is expected to reach 20 million, constituting approximately 22% of the total population of the country.^18^ Since most ocular diseases are attributable to poor knowledge of eye health and poor access to eye health care services in this age group, it is of great importance to determine the prevalence of vision-related abnormalities in this population. A few epidemiological studies have evaluated the prevalence of corneal abnormalities worldwide.^15,19^ The limitation of studies on the prevalence of corneal abnormalities in the elderly population of Iran and the need to examine this issue at different ages along with expectations for an increase in the prevalence of eye diseases due to population aging and lifestyle modification encouraged us to conduct this study to determine the prevalence of corneal abnormalities and their most important risk factors in an Iranian elderly population.

## Materials and Methods

###  Design and Sampling

 The data presented in this report is derived from the Tehran Geriatric Eye Study (TGES), a cross-sectional study carried out in 2019 on individuals aged 60 and above in Tehran, Iran. The study utilized a multi-stage stratified random cluster sampling method. The primary objective of the study was to examine the prevalence of visual impairment among the elderly population in Tehran. To achieve this goal, the sample size was determined based on a 5.2% prevalence rate of visual impairment, with a desired accuracy of 1% and a 95% confidence level. Initially, the sample size was calculated to be 1,894 individuals. However, after accounting for a 1.5 design effect and a 10% non-response rate, the final sample size was adjusted to 3,155 individuals, which was rounded up to 3200 participants.

 In the TGES, a total of 160 clusters were chosen in a random manner, with the selection being proportional to the size of each cluster. These clusters were selected from 22 different strata in Tehran. Once each cluster was determined, a sampling team was dispatched to the corresponding address. The first house located on the southwest side of the chosen block was designated as the cluster head. Subsequently, the neighboring households were selected in a counterclockwise direction. All individuals aged 60 years and above were then invited to take part in the study after being provided with a clear explanation of the study’s objectives and receiving assurance regarding the confidentiality of their data. If an individual expressed their willingness to participate, informed consent was obtained, an identification card was issued, and the person was subsequently transported to Noor Eye Hospital for further examinations. In cases where a household was found to be absent during the initial visit, a follow-up visit was scheduled, preferably in the afternoon on the same day.

 Trained research assistants in the hospital gathered comprehensive demographic, anthropometric, and socioeconomic (SES) information from the participants. Subsequently, the participants underwent optometric and ophthalmologic evaluations. The optometric assessments commenced with refraction utilizing the Nidek ARK-510A auto-refractometer/keratometers (Nidek Co. LTD, Aichi, Japan). Following this, uncorrected (UCVA) and best-corrected (BCVA) visual acuities were assessed using the Smart LC 13 LED visual chart (Medizs Inc., Korea) at a distance of 6 meters. Subsequent examinations of the anterior and posterior ocular segments were conducted utilizing the B900 slit-lamp (Haag-Streit AG, Bern, Switzerland) and a + 90 D lens. Individuals with a history of corneal injury due to trauma or corneal transplant were excluded from the study.

###  Definition of Corneal Abnormalities

 To diagnose corneal abnormalities, the ophthalmologists of this study were first trained to define corneal conditions based on a uniform pattern, and all of the abnormalities were defined based on standard definitions.

*Posterior embryotoxon (PE)*: In this abnormality, evaluation of the posterior peripheral cornea under direct light of the slit-lamp shows a peripheral corneal ring at the margin of Descemet’s membrane displaced closer to the corneal center.^20^

*Punctate epithelial erosion*: This corneal abnormality is a non-specific finding appearing clinically as tiny defects in the epithelium. It is an early sign indicating epithelial compromise that stains positively with fluorescein.^21^

*Pigment on endothelium (POE)*: This abnormality is characterized by corneal endothelial deposits often appearing colored and may be found in the corneal center, periphery, or both.

*Corneal dystrophy*: A bilateral hereditary disorder affecting various corneal layers, such as the epithelium, basal membrane, stroma, and endothelium, presenting as opacities, vesicles, rings, and streaks.^22^

*Corneal vascularization*: This abnormality is characterized by the presence of blood vessels in different corneal layers, particularly the stroma.^23^

*Corneal degeneration*: It is defined as pathological (abnormal) corneal changes during aging.^24^

*Any condition in at least one eye:* This abnormality refers to any condition in at least one eye.

###  Socioeconomic Status

 To assess the socioeconomic status (SES), we collected information on 13 assets owned by households and utilized principle component analysis to create an asset index based on the weights assigned to the first component.

###  Statistical Analysis and Model Building

 The prevalence of corneal anomalies in Tehran was estimated by standardizing the samples based on age and sex according to the 2016 census. The percentage of corneal abnormalities, along with their 95% confidence intervals, was calculated using the exact method. Due to the low prevalence of some corneal abnormalities, Firth’s logistic regression was employed for model building to address sparse data bias.^25^ The relationship between various determinants (such as age, sex, education, employment status, marital status, eye examination, insurance, smoking, alcohol consumption, SES, and outdoor activity) and corneal abnormalities was assessed through simple Firth’s logistic regression analysis. Determinants with a *P *value of less than 0.05 were included in the multiple Firth’s logistic regression analysis. Correction for the cluster effect was applied to standard error, and data analysis was conducted using Stata version 12.0 software, with statistical significance set at *P* < 0.05.

## Results

 Among the 3791 individuals who were invited to participate in the study, a total of 3310 individuals accepted, resulting in a response rate of 87.31%. The average age of the participants was 68.25 ± 6.55 years, ranging from 60 to 97 years, with 57.8% female (n = 1913) and the remaining male. Additionally, 43.56% (n = 1442) were retired, 73.99% (n = 2449) were married, and 12.48% (n = 413) had received a university education.

 The data presented in Table 1 illustrates the age and sex standardized prevalence of various corneal abnormalities. The prevalence rates for PE, PED, POE, CDys, and CV were 0.08% (95% CI: 0.02 to 0.40), 8.77% (95% CI: 6.64 to 11.51), 0.57% (95% CI: 0.33 to 0.98), 0.53% (95% CI: 0.33 to 0.82), and 0.95% (95% CI: 0.60 to 1.52), respectively. Furthermore, the age- and sex-standardized prevalence of any corneal condition in at least one eye was 49.08% (95% CI: 45.90 to 52.26). The prevalence of any corneal condition in at least one eye was observed to increase with age, ranging from 37.52% in the age group of 60 to 64 years to 64.85% in the age group over 85 years.

**Table 1:** Prevalence of Corneal Abnormalities in Tehran Geriatric Eye Study (TGES), 2019

**Variables**		**Prevalence% (95% CI)**						
		**PE**	**PED**	**POD**	**CDys**	**CV**	**CDeg**	**Any Condition in at Least One Eye**
Total*		0.08 (0.02 to 0.40)	8.77 (6.64 to 11.51)	0.57 (0.33 to 0.98)	0.53 (0.33 to 0.82)	0.95 (0.60 to 1.52)	44.87 (41.80 to 47.98)	49.08 (45.90 to 52.26)
Age	60-64 (n = 1166)	0	6.97 (4.74 to 10.13)	0.36 (0.12 to 1.04)	0.39 (0.17 to 0.87)	0.7 (0.25 to 1.94)	34.18 (30.62 to 37.94)	37.52 (34.94 to 40.18)
	65-69 (n = 953)	0.08 (0.01 to 0.61)	8.74 (6.23 to 12.14)	0.67 (0.30 to 1.49)	0.22 (0.05 to 0.92)	0.76 (0.36 to 1.59)	43.05 (38.50 to 47.73)	46.47 (42.55 to 50.43)
	70-74 (n = 632)	0	8.28 (6.03 to 11.28)	0.61 (0.23 to 1.61)	1.03 (0.50 to 2.13)	0.77 (0.32 to 1.85)	52.44 (47.89 to 56.95)	56.42 (50.99 to 61.7)
	74-79 (n = 317)	0	9.44 (5.75 to 15.10)	0.23 (0.03 to 1.62)	1.07 (0.39 to 2.88)	1.01 (0.32 to 3.15)	55.54 (49.77 to 61.17)	60.13 (53.89 to 66.06)
	≥ 80 (n = 242)	0.05 (0.07 to 3.56)	13.75 (8.92 to 20.60)	1.21 (0.38 to 3.82)	0.28 (0.04 to 2.00)	2.20 (0.92 to 5.21)	56.78 (47.77 to 65.35)	64.85 (57.36 to 71.68)
Gender	Male (n = 1397)	0	8.67 (6.02 to 12.33)	0.43 (0.19 to 0.98)	0.48 (0.24 to 0.96)	1.22 (0.65 to 2.26)	48.51 (44.94 to 52.09)	53.43 (49.41 to 57.41)
	Female (n = 1913)	0.17 (0.03 to 0.79)	8.88 (6.60 to 11.84)	0.70 (0.38 to 1.29)	0.57 (0.30 to 1.07)	0.69 (0.35 to 1.37)	41.29 (37.58 to 45.09)	43.81 (40.81 to 46.86)

*Age and sex standardized CI, confidence interval; PE, posterior embryotoxon; PED, Punctate epithelial defect; POD, Pigment on Endothelium; CDys, Corneal Dystrophy; CV, Corneal Vascularization; CDeg, Corneal degeneration.

**Figure 1:**
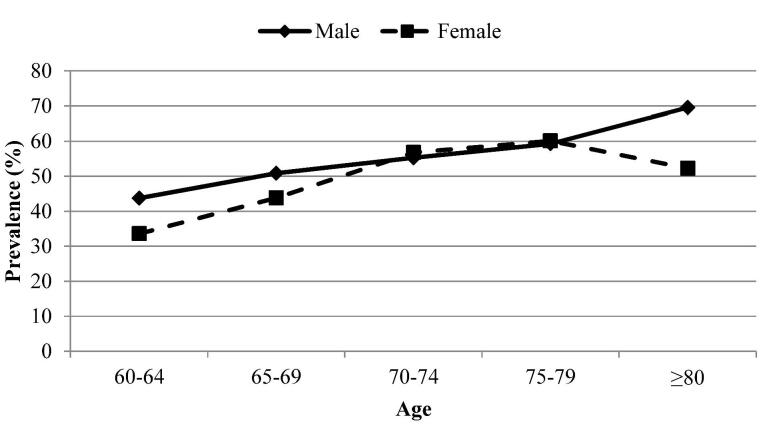
Prevalence of Certain Corneal Conditions in at Least One Eye According to Age and Gender

Figure 1 depicts the prevalence of any corneal condition in at least one eye based on age, categorized by gender.

 The prevalence of corneal degeneration was 44.87%. Among different age groups, individuals aged 80 years and above exhibited the highest prevalence of PED, POE, CV, and corneal degeneration [13.75% (95% CI: 8.92 to 20.60), 1.21% (95% CI: 0.38 to 3.82), 2.20% (95% CI: 0.92 to 5.21), and 56.78% (95% CI: 47.77 to 65.35), respectively]. In terms of gender, women showed the highest prevalence of PED, POE, and CDys [8.88% (95% CI: 6.60 to 11.84), 0.70% (95% CI: 0.38 to 1.29), and 0.57% (95% CI: 0.30 to 1.07), respectively]. The prevalence of any eye condition in at least one eye is detailed based on education and economic status in Table 2. The findings of the multiple logistic regression analysis are presented in Table 3. Among the determinants considered, such as age, sex, education, employment status, marital status, eye examination, insurance, smoking, alcohol consumption, SES, and outdoor activity, only some met the criteria for inclusion in the multiple model. Notably, no models were developed for CDys and PE as none of the determinants met the criteria for inclusion in the multiple model.

**Table 2:** Prevalence of Corneal Abnormalities in Tehran Geriatric Eye Study by Education and Socioeconomic Status

**Variables**		**% (95% CI)**
Education	Illiterate	54.55 (48.18-60.92)
	Primary School	47.78 (42.29-53.27)
	Guide School	48.14 (43.73-52.55)
	High School	46.73 (41.99-51.48)
	College	51.55 (45.67-57.43)
Socioeconomic status	Low	52.92 (48.09-57.75)
	Mid	49.39 (43.22-55.56)
	High	48.77 (44.44-53.10)
	Highest	43.59 (38.58-48.60)

**Table 3:** Association Between Corneal Abnormalities with Varies Determinants Based on Multiple Firth's Logistic Regression in Tehran Geriatric Eye Study (TGES), 2019

	**Variables**		**OR (95% CI)**	***P*** ** Value**
Punctate epithelial defect	Age (baseline: 60 years old)	65-69	1.27 (0.92 to 1.75)	0.147
		70-74	1.17 (0.81 to 1.69)	0.380
		75-79	1.34 (0.86 to 2.11)	0.180
		> 80	2.03 (1.32 to 3.16)	0.001*
	SES (baseline: Lowest)	Low	0.71 (0.49 to 1.07)	0.082
		Mid	0.73 (0.48 to 1.10)	0.130
		High	0.67 (0.42 to 1.02)	0.061
		highest	0.52 (0.33 to 0.81)	0.004*
Pigment on Endothelium	Education (baseline: illiterate)	Primary school	0.23 (0.07 to 0.72)	0.012*
		Guide school	0.29 (0.08 to 1.03)	0.056
		High school	0.03 (0.01 to 0.54)	0.017*
		Collage	0.44 (0.12 to 1.52)	0.194
Corneal Vascularization	Age (baseline: 60 years old)	65-69	1.27 (0.44 to 3.65)	0.663
		70-74	1.35 (0.42 to 4.29)	0.615
		75-79	1.52 (0.40 to 5.85)	0.541
		> 80	3.74 (1.15 to 12.11)	0.028*
	SES (baseline: Lowest)	Low	0.31 (0.11 to 0.91)	0.033*
		Mid	0.55 (0.19 to 1.59)	0.269
		High	0.17 (0.04 to 0.72)	0.017*
		highest	0.43 (0.13 to 1.41)	0.165
	Sex (baseline: Male)		0.44 (0.20 to 0.99)	0.048*
Corneal degeneration	Age (baseline: 60 years old)	65-69	1.42 (1.19 to 1.71)	< 0.001*
		70-74	2.13 (1.74 to 2.62)	< 0.001*
		75-79	2.24 (1.72 to 2.92)	< 0.001*
		> 80	2.79 (2.06 to 3.78)	< 0.001*
	SES (baseline: Lowest)	Low	1.02 (0.8 to 1.31)	0.859
		Mid	1.05 (0.81 to 1.38)	0.701
		High	1.02 (0.78 to 1.35)	0.878
		Highest	0.81 (0.6 to 1.09)	0.160
	Education (baseline: illiterate)	Primary school	0.86 (0.68 to 1.09)	0.206
		Guide school	0.82 (0.63 to 1.07)	0.139
		High School	0.97 (0.75 to 1.27)	0.833
		Collage	1.29 (0.93 to 1.79)	0.134
	Employment (baseline: Employed)	Retired	2.04 (1.32 to 3.16)	0.001*
		housekeeper	2.72 (1.65 to 4.5)	< 0.001*
		other	1.96 (1.13 to 3.38)	0.016*
	Smoking (baseline: No)		1.05 (0.84 to 1.31)	0.681
	Sex (baseline: Male)		0.57 (0.44 to 0.76)	< 0.001*

SES, socioeconomic status; NN, No Need. *Significance. None of the determinants has any significant association with Corneal dystrophy and Posterior Embryotoxon.

 Based on the findings presented in Table 2, PED exhibited a positive correlation with age (OR: 2.03 for individuals over 80 years old compared to those aged 60-64 years, *P* = 0.001) and a negative relationship with SES (OR: 0.52 for the highest SES group compared to the lowest, *P* = 0.004). Conversely, corneal degeneration showed a direct association with age (OR: 2.79 for individuals over 80 years compared to those aged 60-64 years; *P* < 0.001) and an inverse correlation with female gender (OR: 0.57, *P* < 0.001). Additionally, employment status was significantly linked to corneal degeneration (OR: 2.04 for retired individuals versus employed, *P* = 0.001; OR: 2.72 for housekeepers versus employed, *P* < 0.001; OR: 1.96 for others versus employed, *P* = 0.016). Female gender was negatively associated with corneal degeneration (OR: 0.57, *P* < 0.001).

 In relation to the outcomes, CV demonstrated a positive connection with age (OR: 3.74 for individuals over 80 years compared to those aged 60-64 years, *P* = 0.028) and negative relationships with SES (OR: 0.17 for high SES group compared to the lowest, *P* = 0.017) and female gender (OR: 0.44, *P* = 0.048).

 The average best corrected visual acuity in eyes with at least one abnormality was 0.17 ± 0.51, while eyes without any problems had a mean value of 0.16 ± 0.43 logMAR (*P* = 0.748).

## Discussion

 The TGES was the first Iranian population-based study exclusively conducted on subjects aged 60 years and over living in Tehran, capital of Iran. This study, as part of the TGES, was performed to determine the prevalence of corneal abnormalities according to age, sex, and other demographic characteristics to shed light on their risk factor; therefore, its results can help to better understand the epidemiologic profile of corneal abnormalities.^15^

 An extensive literature search showed only one population-based study on the prevalence of cornmeal abnormalities^13^ and the majority of the studies in this regard are clinic-based or medical records-based. According to the results, 50% of the study population had at least one of the corneal abnormalities, which was higher than the prevalence reported by Hashemi et al.^13^ Moreover, the most common abnormality in the present study was corneal degeneration with a prevalence of 44.87%, while in the study by Hashemi et al, the most common abnormality was PE with a prevalence of 14.7%.^13^

 Although few studies have investigated corneal abnormalities in the world and Iran, caution should be practiced when comparing their results due to differences in the age structure and design.^20,26,27^ The results of the present study showed that less than 0.1% of the participants had PE, which was much lower than previous studies; for example, Hashemi et al reported a prevalence of 14.7% for PE in the Shahroud Eye Cohort Study.^13^ Clinic-based studies have also reported a higher prevalence compared to the present study; for example, the prevalence of PE was 6.8% in a study by Rennie et al,^20^ 15% in a study by Burian et al,^28^ 24.3% in a study by Ozeki et al,^27^ and 95% in subjects suffering from Alagille syndrome.^26^ What is clear is that the difference in the prevalence of PE between this study and the study conducted by Hashemi et al^13^ is due to age structure since the age range of the participants was 40-65 years in the above study and 60-97 years in the present study. It becomes more difficult to observe and detect PE with an increase in age due to increase in other corneal degenerative diseases and peripheral corneal opacity including arcus senilis,^20^ which may be a reason for the lower prevalence of this corneal opacity in the present population compared to another study.^16^ On the other hand, the prevalence of PE was lower in our study compared to other clinic-based studies, which could be due to diagnosis based on histology in those studies^20,26,27^ which is more accurate than diagnosis based on slit lamp examination.

 Although CDys is one of the most important reasons for corneal transplant in the world, especially among children, ^6,29^ less than 1% of the participants had CDys and this abnormality had the lowest prevalence among all corneal abnormalities in the present study. In line with the results of this study, Musch et al evaluated all records available in the national managed-care network and reported a prevalence of less than 1% in the USA.^15^ Similarly, Hashemi et al also reported a prevalence of less than 1% in the Shahroud Eye Cohort Study.^13^ According to other studies, the prevalence of CDys is 11% in individuals aged 50 years and over,^30^ 4.5%-9% in Europe,^31^ and 3.8-4.1% in Asia.^19,32^ It is clear that genetics play the most important role in CDys^15,33^; however, previous studies found that personal factors also increased its occurrence and worsened its manifestations.^31^ Although some studies reported that the cumulative effect of environmental factors can result in increased prevalence of CDys in the elderly,^34^ this relationship was not observed in the present study. To explain this finding, it should be noted that CDys can be usually detected with a slit lamp after 30 years of age, and the chance of detection increases with age. Therefore, its high prevalence in advanced ages is related to the higher chance of diagnosis. In other words, age is not a causative factor but is a proxy for detectability.

 Moreover, in the present study, the prevalence of CDys was higher in women but the difference was not significant, which could be due to differences in the study population (ethnicity), diagnostic tool, and diagnostic accuracy of the tool used for diagnosis.^35^ Other studies have also reported a higher prevalence of CDys in women,^13,15,31^ which could be mostly due to genetic differences between men and women. However, this difference may be in part due to hormonal differences and environmental factors that affect its occurrence and manifestations.^36^ Zoega et alfound that smoking increased the chance of CDys by more than twice.^31^ In the present study, the prevalence of CDys was higher in smokers versus non-smokers (data not shown) but the difference was not significant.

 The prevalence of PED was 8.77% in the present study. According to some reports, about 200 000 cases of PED occur in the USA every year,^37^ comprising more than 10% of all eye-related emergency room visits.^38^

 The prevalence of CV was about 1% in the present study, which was higher than the value reported by Hashemi et al.^13^ The reason for this disparity may be the age difference between the two studies. However, the prevalence of CV was largely affected by age, such that it increased from 0.7% in individuals aged 60-64 years to 2.2% in participants aged 80 years and over in the present study. According to the results of the multiple logistic model, age had a direct relationship, and economic status had an indirect relationship with the prevalence of PED and CV, which is consistent with other studies.^13^ Considering the findings of other studies, an increase in age can predict an increase in corneal abnormalities including PED and CV.^13,20,27^ It should be borne in mind that different factors such as trauma, infectious diseases, UV exposure, MGD, dry eye, and malnutrition including vitamin A deficiency are effective in the occurrence of CV and PED and age and economic status may serve as proxies for these factors.

 As mentioned earlier, corneal degeneration was the most common corneal abnormality, comprising 91% of all cases excluding corneal opacity. It should be noted that corneal degeneration includes a number of abnormalities like fatty degeneration and arcus senilis, all of which have a direct relationship with age^39^ and can be a reason for the higher prevalence of this abnormality in this age range. The present study found a direct relationship between corneal degeneration. This is line with the literature that other ocular disorders have a strong association with age.^39^

 Another finding of the present study was the role of gender in corneal abnormalities. The results of the present study show that there is an inter-gender difference in the prevalence of CV and corneal degeneration in such a way that the chance of these abnormalities was lower in women. Studies have rarely discussed the reason for this difference; however, it has been shown that women are less susceptible to disorders affecting corneal changes (like dry eye) due to hormonal differences,^3^ which could be a reason for the lower prevalence of CV and degeneration in women.

## Conclusion

 In summary, it can be deduced that half of the individuals who are 60 years old and above possess at least a single corneal abnormality. PE was the rarest, and corneal degeneration was the most common corneal abnormality, comprising 91% of all cases. According to the results of model building, age, economic status, education level, and sex were the determinants of the occurrence of corneal abnormalities, among which age was the most important determinant. Age stands out as the primary factor influencing the development of corneal abnormalities. Consequently, special attention should be directed towards the elderly population when formulating strategies for diagnosis and treatment. It should be noted that the results of the present study are related to the city of Tehran, which has a heterogeneous population in terms of race and ethnicity, and its results can be generalized to all of Iran. Moreover, the present study was conducted on people aged 60 years and above. There is a limitation of comparison with this age group. However, due to the lack of studies in this field, the results of this study are valuable. Selection bias and measurement bias may affect the results; however, these errors are minimized due to stratified cluster random sampling and the fact that the definition and protocol for determining corneal problems are the same for everyone.
